# Einsatz von Instrumenten bei der klinischen Untersuchung in deutschen HNO-Abteilungen und Privatpraxen heutzutage

**DOI:** 10.1007/s00106-021-01096-z

**Published:** 2021-08-17

**Authors:** Achim M. Franzen, Hannes Sykora, Michael Hauptmann, Annekatrin Coordes

**Affiliations:** 1 Campus Ruppiner Kliniken, Klinik für HNO-Krankheiten und Plastische Operationen, Medizinische Hochschule Brandenburg Theodor Fontane, Neuruppin, Deutschland; 2 Institut für Biometrie und Registerforschung, Medizinische Hochschule Brandenburg Theodor Fontane, Neuruppin, Deutschland; 3 Fakultät für Gesundheitswissenschaften, Gemeinsame Fakultät der Brandenburgischen Technischen Universität Cottbus – Senftenberg, der Medizinischen Hochschule Brandenburg Theodor Fontane und der Universität Potsdam, Senftenberg, Deutschland; 4 Klinik für Hals-, Nasen- und Ohrenheilkunde, Campus Virchow Klinikum and Campus Charité Mitte, Charité – Universitätsmedizin Berlin, Corporate Member der Freien Universität Berlin and Humboldt-Universität zu Berlin, Augustenburger Platz 1, 13353 Berlin, Deutschland

**Keywords:** Hals-Nasen-Ohren-Heilkunde, Stirnspiegel, Mikroskop, Endoskop, Otoskop, Otorhinolaryngology, Forehead mirror, Microscope, Endoscope, Otoscope

## Abstract

**Hintergrund:**

Der klassische Stirnreflektor diente dem HNO-Arzt zur Spiegeluntersuchung und ist heute Mediensymbol für den Arzt. Mit welchen Instrumenten heute in Deutschland HNO-Patienten klinisch untersucht werden, ist nicht bekannt. Es ist daher das Ziel der vorgelegten Untersuchung, dies mithilfe einer Befragung zu ermitteln.

**Material und Methoden:**

Es erfolgte die Auswertung von 321 Fragebögen von klinisch tätigen (172) und niedergelassenen (149) HNO-Ärzten.

**Ergebnisse:**

Die HNO-Spiegeluntersuchung wird heute mit einer selbstleuchtenden Kopflampe mit Akku und/oder Lichtleitkabel durchgeführt. Etwa 20 % der Antwortenden verwendet auch einen Stirnspiegel. Das Mikroskop wird von 90 % der teilnehmenden HNO-Ärzte zur Untersuchung der Ohren eingesetzt. Ein starres Endoskop benutzen 53,3 % zur Untersuchung des Kehlkopfs, 41 % für den Epipharynx und 35 % für die Nase/Nasennebenhöhlen (34,6 %). Flexibles Endoskop und Otoskop werden lediglich fakultativ verwendet.

**Schlussfolgerung:**

Die selbstleuchtende, in den neuen Bundesländern häufiger kabellose Kopflampe hat den klassischen Stirnreflektor, mit dem seit ca. 20 Jahren nicht mehr ausgebildet wird, weitgehend verdrängt. Mit großer Regelmäßigkeit werden zumindest einzelne Organe auch mit dem Mikroskop oder starren Endoskop untersucht, während das flexible Endoskop und Otoskop insgesamt viel seltener, vor allem von Jüngeren und im Krankenhaus Tätigen, verwendet werden. Das diagnostische Potenzial der flexiblen Endoskopie wird durch die ambulanten Vergütungsstrukturen in Deutschland möglicherweise kompromittiert.

## Abstract (en)

**Background:**

The classical forehead reflector as traditionally used by ear, nose, and throat (ENT) physicians for the ENT examination is now iconic for doctors in general. It is unknown which instruments are currently used in Germany to clinically examine ENT patients. Therefore, this study aims to present results of a survey about commonly used instruments.

**Materials and methods:**

An evaluation of 321 questionnaires from ENT doctors working in general and university hospitals (172) and in private practices (149) was performed.

**Results:**

The ENT mirror examination is nowadays carried out with a self-illuminating headlamp with battery and/or light guide cable. Approximately 20% of respondents also use a forehead mirror. The microscope is used by 90% of doctors to examine the ears; a rigid endoscope was used in 53.3% to examine the larynx, epipharynx (41.1%), and the nose/sinuses (34.6%). Flexible endoscopes and otoscopes are used only rarely.

**Conclusion:**

The self-illuminating headlamp, which is more often wireless in eastern Germany, has largely replaced the classical forehead reflector, with which doctors younger than 40 years were no longer trained. At least some organs are also examined very regularly with the microscope or rigid endoscope. The flexible endoscope and otoscope are used much less frequently overall, mainly by younger physicians and ENT doctors working in hospitals. The diagnostic potential of flexible endoscopy may be compromised by the outpatient remuneration structures in Germany.

## Hintergrund und Fragestellung

**Figure 1:**
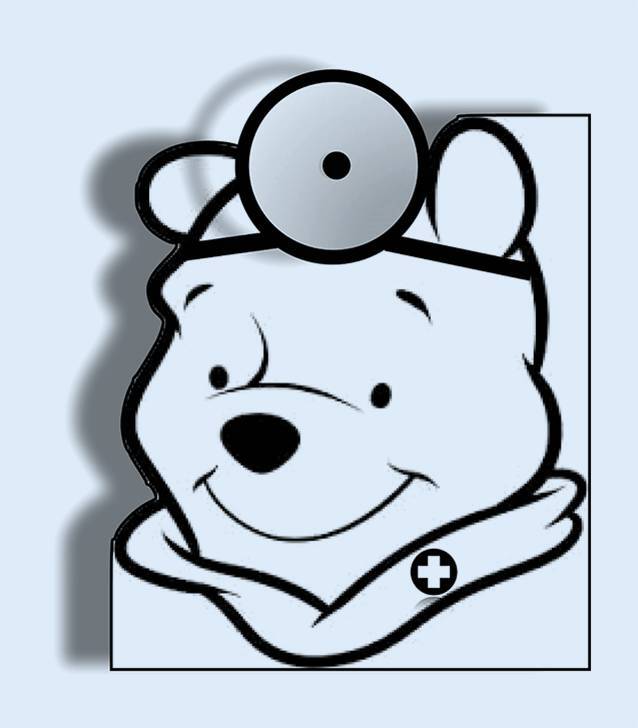

Die Organe des oberen Aerodigestivtrakts gehören exemplarisch zu den schwer zugänglichen Körperregionen, deren Beurteilung besondere Untersuchungsinstrumente erforderlich machen. Von großer Bedeutung für die Hals-Nasen-Ohren-Heilkunde war die erste Beschreibung eines „Hohlspiegels mit zentraler Durchlöcherung“ durch Friedrich Hofmann, Burgsteinfurt, im Jahr 1841, der um 1855 durch von Tröltsch zunächst für die Otoskopie etabliert wurde [5]. Der Hohlspiegel war ein ideales Instrument zur Beleuchtung und gleichzeitigen parallaxenfreien Betrachtung. Zunächst waren das Tageslicht oder eine Kerze die erforderlichen Lichtquellen, später wurde elektrisches Licht verwendet. Die über die Grenzen der Medizin hinausgehende Bedeutung des Stirnspiegels wird auch dadurch verdeutlicht, dass der verkehrt herum getragene, nach oben geklappte Spiegel in diversen Medien als Symbol des Arztes weit verbreitet ist (Abb. 1; [10]).

Als Alternative zu dem an einem Stirnreif vor einem Auge fixierten Stirnspiegel wanderte später die Lichtquelle an eine zentrale Position in der Stirnmitte [11].

Ab den 1920er-Jahren stehen außerdem stereoskopische Mikroskope, ab den 1960er-Jahren nach der Entwicklung von Stablinsenoptiken starre Kaltlichtendoskope und später auch flexible, auch für die Passage durch die Nase geeignete Endoskope für die klinische Untersuchung von HNO-Patienten zur Verfügung [8].

Mit welchen Instrumenten heute angesichts der zahlreichen Optionen Patienten in der Praxis und im Krankenhaus untersucht werden, ist nicht bekannt. Es ist das Ziel der vorgelegten Untersuchung, dies auf dem Weg einer Befragung zu ermitteln. Außerdem sollen ggf. bestehende Unterschiede, z. B. zwischen den Generationen, zwischen Klinik und Praxis oder innerhalb Deutschlands detektiert werden. Von ausdrücklichem Interesse ist zuletzt auch die Frage, welche Rolle der Stirnreflektor in der aktuellen Praxis noch spielt.

## Studiendesign und Untersuchungsmethoden

**Figure 2:**
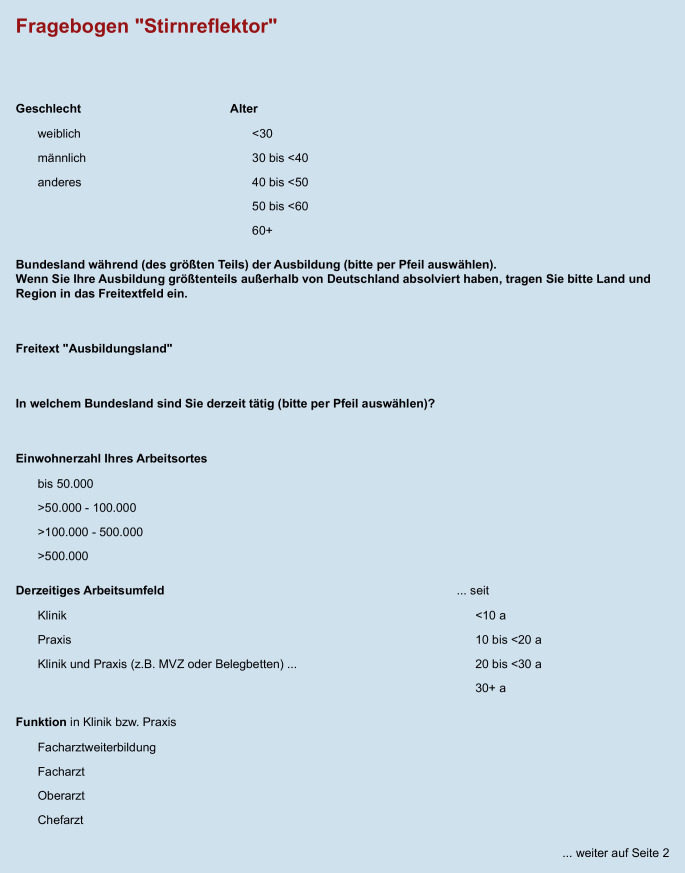

**Figure 3:**
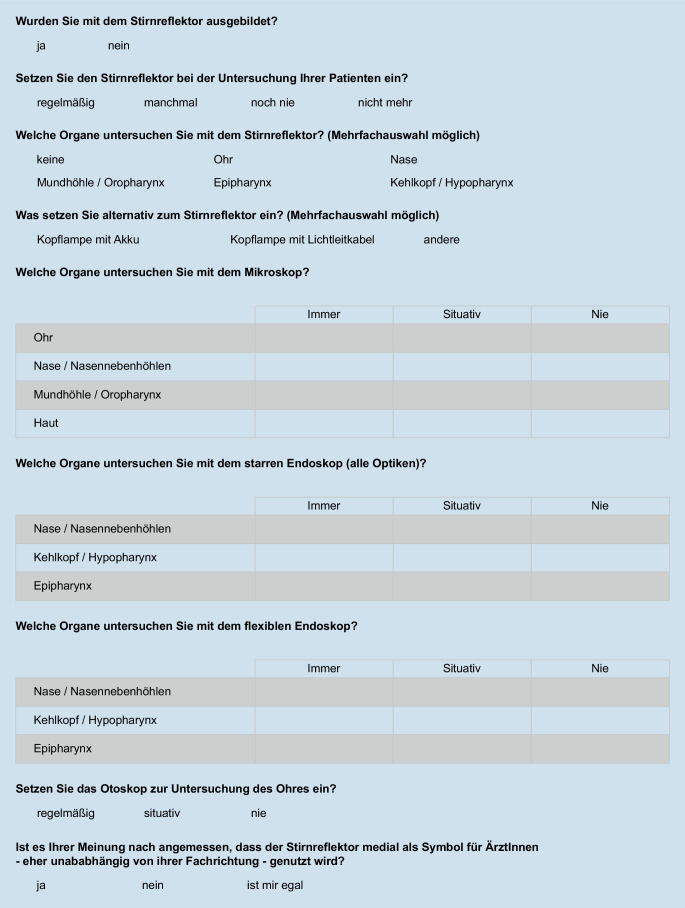

Der von uns entwickelte und im Herbst 2019 versandte Fragebogen (Abb. 2, Abb. 3) umfasste 18 Fragen zur Person (u. a. Alter in Dekaden, Geschlecht, Ausbildungs- und derzeitiges Tätigkeitsumfeld) und zu Untersuchungsmethoden und eingesetzten Geräten (Stirnreflektor, Stirnlampe, Mikroskop, starres/flexibles Endoskop, Otoskop). Explizit gestellt wurde die Frage nach der Ausbildung mit dem Stirnreflektor; die Intensität der Nutzung der einzelnen Instrumente konnte differenziert werden (immer, situativ, nie).

Adressaten der Befragung waren niedergelassene und klinisch tätige HNO-Ärzte. Zur Weiterleitung der Fragebögen an niedergelassene HNO-Ärzte nahmen wir Kontakt zum Deutschen HNO-Berufsverband (Organisationsgrad ≥ 90 % der niedergelassenen Ärzte) auf, zur Weiterleitung an die in 160 HNO-Hauptabteilungen und 35 Universitätsklinika klinisch tätigen HNO-Ärzte kontaktierten wir die Deutsche Gesellschaft für HNO-Heilkunde, Kopf- und Hals-Chirurgie.

Zur Untersuchung des Einflusses der Region Deutschlands, in der die Befragten ausgebildet wurden bzw. derzeit tätig sind, etablierten wir drei Untergruppen: Stadtstaaten, neue Bundesländer und alte Bundesländer. Relevante Daten der Bundesärztekammer und der Kassenärztlichen Bundesvereinigung wurden soweit verfügbar abgefragt.

Die vorwiegend deskriptive statistische Auswertung der Daten wurde mit SPSS Statistics 23 (Statistical Package for the Social Sciences, IBM®, Armonk, NY, USA) vorgenommen. Neben dem Darstellen univariater Häufigkeiten wurden Assoziationen zwischen kategorialen Variablen mit dem Chi-Quadrat-Test analysiert.

## Ergebnisse

Insgesamt 321 vollständig/auswertbar beantwortete Fragebögen erhielten wir von 172 in einer Klinik und 149 niedergelassenen oder in einem MVZ (Medizinisches Versorgungszentrum) tätigen Ärzten. Die Fragebögen wurden aus unterschiedlichen Gründen (z. B. Datenschutz) in lediglich 8/18 Landesverbänden an die Mitglieder des Berufsverbands versandt (39,5 % aller Mitglieder) [2]. Auch über die Deutsche HNO-Gesellschaft konnten die Fragebögen direkt lediglich an die Leiter der HNO-Hauptabteilungen und Universitätsklinika – mit der Bitte um Weiterleitung an die nachgeordneten Mitarbeiter – versandt werden. Wir schätzen, dass insgesamt ungefähr 1350 HNO-Ärzte die Einladung zur Befragung direkt erhalten haben. Die Response-Rate wird deshalb mit ca. 321/1350 (24 %) geschätzt.

**Figure 4:**
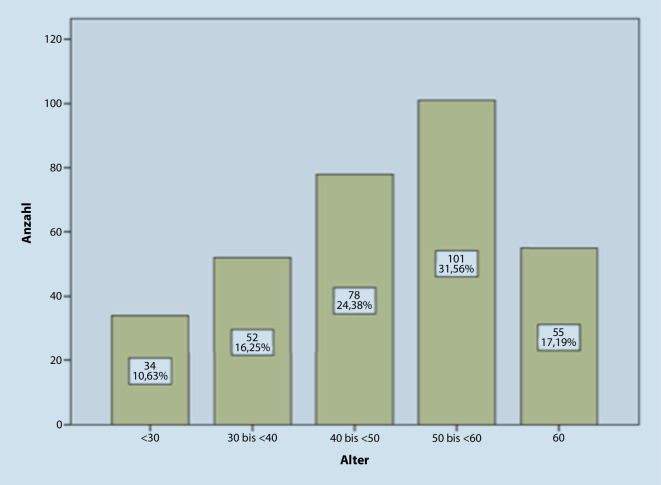

An der Befragung nahmen 110 (34,4 %) Frauen und 209 (65,4 %) Männer teil. Andere und keine Angabe machten zwei (0,6 %). Die am stärksten vertretene Altersgruppe war die der 50- bis 60-Jährgen (Abb. 4). Von den 172 Klinik- und 149 niedergelassenen Ärzten waren 10,2 % in Stadtstaaten (Berlin, Hamburg), 45,4 % in den neuen Bundesländern (vor allem Brandenburg und Sachsen) und 44,4 % in den alten Bundesländern (vor allem Bayern und Nordrhein-Westfalen) tätig. Die Verteilung der Bundesländer, in denen die Befragten ausgebildet wurden, war mit 11,3 %, 45,8 % bzw. 42,4 % ähnlich. Darüber hinaus wurden 13 HNO-Ärzte (4 %) außerhalb Deutschlands, vornehmlich in Osteuropa, ausgebildet. Zum Zeitpunkt der Befragung waren 267 (83,2 %) als Fachärzte tätig, 54 als Ausbildungsassistenten. Unter den niedergelassenen Ärzten lag der Anteil der Fachärzte bei 98 %; unter den 172 Klinikärzten waren 59 Chefärzte und 62 Oberärzte.

213 Befragte (66,4 %) gaben an, mit dem Stirnreflektor ausgebildet worden zu sein (Tab. 1). Von den 13 im Ausland Ausgebildeten (11 jünger als 40 Jahre) wurden 11 (85 %) mit dem Stirnreflektor ausgebildet. 66 Befragte (20,5 %) gaben an, den Reflektor auch aktuell zumindest manchmal einzusetzen, insbesondere zur Untersuchung von Mundhöhle/Oropharynx (65/66) und Nase (56/66). Aktuelle Nutzer eines Stirnreflektors sind vor allem > 60-jährige Fachärzte (47 % dieser Gruppe), etwas häufiger weiblich und doppelt so häufig in einer Praxis tätig wie in einer Klinik (30 % vs. 15 %).

**Table 1:** 

	Immer Anzahl* n *(%)	Situativ Anzahl* n *(%)	Nie Anzahl* n *(%)
*Mikroskop*			
Ohr	289 (90,3 %)	28 (8,8 %)	3 (0,9 %)
Nase/Nasenebenhöhlen	43 (13,9 %)	213 (69,7 %)	54 (17,4 %)
Mundhöhle/Oropharynx	30 (9,7 %)	214 (69,3 %)	65 (21 %)
Haut	48 (15,1 %)	239 (75,4 %)	30 (9,5 %)
*Starres Endoskop*			
Nase/Nasennebenhöhlen	111 (35,1 %)	201 (63,6 %)	4 (1,3 %)
Kehlkopf/Hypopharynx	171 (53,6 %)	138 (43,3 %)	10 (3,1 %)
Epipharynx	132 (41,5 %)	170 (53,5 %)	16 (5 %)
*Flexibles Endoskop*			
Nase/Nasennebenhöhlen	28 (9,2 %)	225 (74,3 %)	50 (16,5 %)
Kehlkopf/Hypopharynx	36 (11,4 %)	257 (81,1 %)	24 (7,6 %)
Epipharynx	37 (11,7 %)	251 (79,7 %)	27 (8,6 %)
*Otoskop*	11 (3,4 %)	121 (37,9 %)	187 (58,6 %)

Zur Untersuchung ihrer Patienten setzten 217 Befragte eine Kopflampe mit Akku und 205 mit einem Lichtleitkabel ein, 101 benutzten beide Varianten; in den neuen Bundesländern war der Anteil derer, die (auch) eine kabellose Lampe benutzten, höher (74,4 % vs. 61,6 %).

Das Mikroskop wird von 289 (90 %) HNO-Ärzten bei jedem Patienten zur Untersuchung der Ohren eingesetzt. Andere Organe werden in weniger als 15 % immer mit einem Mikroskop untersucht; situativ setzen die Befragten das Mikroskop zur Untersuchung von Nase/Nasennebenhöhlen und Mund‑/Rachenraum in 2/3 der Fälle und zur Untersuchung der Haut in ca. 75 % der Fälle ein.

Ein starres Endoskop mit unterschiedlichen Winkeloptiken verwenden 53,3 % der teilnehmenden HNO-Ärzte immer zur Untersuchung des Kehlkopfs, 41,1 % zur Untersuchung des Epipharynx und 34,6 % bei Nase/Nebenhöhlen. In allen übrigen Fällen wird das Gerät zumindest situativ verwandt.

Das flexible Endoskop verwenden nur 10 % der Befragten bei jedem Patienten, zwischen 70 % (Nase/Nasennebenhöhlen) und 80 % (Kehlkopf) setzen das Gerät situativ ein. Während ca. 20 % der in der Praxis tätigen niemals ein flexibles Endoskop nutzen, tun dies 15 %, insbesondere jüngere Kollegen in der Klinik bei jedem Patienten.

Das Otoskop wird von HNO-Ärzten in Klinik und Praxis lediglich von 3,4 % immer eingesetzt und von 58,3 % nie; die übrigen (38,3 %) nutzen es situativ. 70 % der niedergelassenen HNO-Ärzte verwenden das Otoskop nie; unter den klinisch tätigen Ärzten sind es vor allem die jüngsten, die das Otoskop situativ einsetzen (55,9 %), während ca. 65 % der über 50-Jährigen Otoskope nie verwenden.

Der Umstand, dass der Stirnreflektor auch aktuell noch als Symbol für (HNO-)Ärzte verwandt wird, ist den meisten Befragten egal (45,8 %) oder stößt insbesondere bei den < 30-Jährigen sogar auf Ablehnung (24,6 %).

## Diskussion

Wie HNO-Ärzte in deutschen Kliniken und Praxen ihre Patienten klinisch untersuchen, also unter Einsatz welcher Untersuchungsmethoden und Geräte heute eine „Spiegeluntersuchung“ stattfindet, ist nicht bekannt. Die durchgeführte Literaturrecherche machte außerdem deutlich, dass dies auch international kaum anders aussieht [11].

Im Rahmen der von uns durchgeführten Untersuchung werteten wir 321 beantwortete Fragebögen aus den neuen und alten Bundesländern und Stadtstaaten aus. Die Altersverteilung der antwortenden Kollegen mit einem Maximum bei den 50- bis 60-Jährigen entsprach exakt der tatsächlichen Verteilung in Deutschland. Die Antwortenden waren überwiegend als Fachärzte (82 %) entweder in der Praxis oder in der Klinik tätig. Aus den Kliniken erhielten wir überrepräsentativ viele Antworten von leitenden Ärzten.

Die klinische HNO-Untersuchung (Spiegeluntersuchung) wird in allen deutschen HNO-Einrichtungen mit einer fokussierbaren Stirnlampe durchgeführt; in den neuen Bundesländern häufiger mit einem System ohne Kabel. Lediglich ca. 20 % der Antwortenden untersuchen heute vor allem Nase und Mundhöhle mit dem Stirnreflektor. Wurden nach unseren Ergebnissen die > 40-Jährigen überwiegend noch mit dem Stirnreflektor ausgebildet, gilt dies für jüngere nicht mehr! Die Antworten von insbesondere in Osteuropa ausgebildeten Ärzten deuten an, dass es international Unterschiede geben könnte. Das Schicksal des Stirnspiegels für die klinische HNO-Untersuchung in Deutschland scheint also besiegelt.

Im Unterschied dazu steht die aktuelle Präsenz des Stirnreflektors in primär nichtmedizinischen Medien als das verbreitetste Symbol des Arztes überhaupt [4]. Dieser Aspekt hat für die Befragten überwiegend keine Bedeutung oder stößt, häufiger bei jüngeren, auf Ablehnung.

Nahezu alle Patienten werden sowohl in Kliniken als auch in Praxen über die Spiegeluntersuchung hinaus auch mit einem Mikroskop und/oder Endoskop untersucht.

Dies gilt vor allem für die Untersuchung der Ohren, die nahezu bei jedem Patienten mit dem Mikroskop untersucht werden. Bei anderen Organen wird das Mikroskop nur selten regelmäßig angewendet, in einem nennenswerten Umfang allerdings fakultativ. In diesem Kontext nennen die meisten Befragten die Beurteilung von Hautveränderungen. Auch im Fachgebiet Dermatologie spielt das Mikroskop bekanntlich im Rahmen der Tumordiagnostik eine große Rolle. Insbesondere zur regelmäßigen Untersuchung von Nase/Nasennebenhöhlen wird das Mikroskop seltener verwendet als das Endoskop; der erhöhte Aufwand für die Reinigung des Endoskops scheint bei der Wahl nicht ins Gewicht zu fallen. Auch die Untersuchung des Kehlkopfs mit Mikroskop in Verbindung mit einem Spiegel ist möglich, wurde allerdings von uns nicht ausdrücklich abgefragt. Bei einer Auswertung der Abrechnungsdaten der US-amerikanischen Krankenversicherung „Medicare“ wurden mikroskopische Ohruntersuchungen in Verbindung mit einer Zerumenentfernung bei Weitem am häufigsten abgerechnet [6].

Das in der Häufigkeit des Einsatzes nach dem Mikroskop folgende Instrument ist das starre Endoskop mit unterschiedlichen Winkeloptiken, das bei mehr als jedem zweiten Patienten zur Untersuchung des Kehlkopfs eingesetzt wird. Regelmäßig werden auch Epipharynx (> 40 %) und Nase/Nasennebenhöhlen (ca. 35 %) so untersucht. Darüber hinaus berichten alle Antwortenden, starre Endoskope zumindest situativ zu verwenden. Die Bedeutung der starren Lupenlaryngoskopie für die Beurteilung des Situs und im Rahmen einer Stroboskopie, gute Untersuchungsbedingungen vorausgesetzt, ist sicher evident. Die Bedeutung der endoskopischen Untersuchung im Vergleich zur klassischen anterioren Rhinoskopie konnte vor allem bei anatomischen Veränderungen wie z. B. einer Septumdeviation, aber auch bei einem Vergleich mit einer CT demonstriert werden [9]. Hur et al. [6] fanden eine Steigerung der durch niedergelassene US-amerikanische Ärzte durchgeführten Nasenendoskopien um 313 % zwischen 2000 und 2016. Ein Zusammenhang zwischen den erheblichen regionalen Schwankungen und der Dichte der HNO-Ärzte, der Zahl der Versicherten und der Höhe der Vergütung wurde vermutet. Im Unterschied dazu nutzten in einer 1989 publizierten Studie aus England nur ca. 10 % der befragten niedergelassenen HNO-Ärzte starre Winkeloptiken zur Untersuchung von Nasopharynx und Larynx; insbesondere die Laryngoskopie betreffend wird dieses Verhalten damit erklärt, dass die starre Endoskopie weder hinsichtlich des Würgereizes noch der Übersicht (überhängende Epiglottis) Vorteile gegenüber der Untersuchung mit dem Spiegel hat [1].

Letztgenannte Aspekte betreffend stellt das flexible Endoskop eine wirkliche Alternative dar, wird aber nach unseren Ergebnissen nur sehr selten bei jedem Patienten eingesetzt; auch unter Berücksichtigung eines situativen Einsatzes wird deutlich, dass in der Praxis tätige Kollegen das flexible Endoskop seltener und jüngere Kollegen (in der Klinik) es häufiger nutzen. Im Unterschied hierzu ist die flexible Laryngoskopie die zweithäufigste Prozedur, die niedergelassenen HNO-Ärzte in den USA aktuell abrechnen. Zwischen den Jahren 2000 und 2016 wurde ein Anstieg der durchgeführten Prozeduren um 87 % festgestellt [7].

Ursache für den seltenen Einsatz vor allem flexibler Endoskope in deutschen HNO-Praxen könnte ein Missverhältnis von Kosten und Erlösen sein. Mit der Novellierung der Abrechnung ambulanter Leistungen (EBM 2000Plus) wurde die Einzelleistungsvergütung weitgehend durch eine Pauschale ersetzt. Aktuell wird lediglich die Lupenlaryngoskopie mit 74 Punkten (8,23 €) vergütet; weitere mikroskopische und endoskopische Untersuchungen einschließlich der flexiblen Rhinolaryngoskopie sind in der Konsultationspauschale enthalten. Ein weiterer betriebswirtschaftlicher Aspekt ist die im EBM 2000Plus fehlende Vergütung für die Aufbereitung von Medizinprodukten, die seit der Einführung des Medizinproduktegesetzes, der Medizinprodukte-Betreiberverordnung etc. mit einem erheblichen Aufwand (und Kosten) verbunden ist.

In den USA wurde die flexible Rhinolaryngoskopie im Jahr 2016 als Einzelleistung mit 85 bis 104 US-$ vergütet. Bereits in den 1980er-Jahren wurde mehr als jeder zweite ambulante Patient in England (auch) mit einem flexiblen Rhinolaryngoskop untersucht. Als Begründung werden nicht nur die gute Übersicht über praktisch jeden Kehlkopf bei einem wachen Patienten benannt, sondern vor allem die eben auch kostenrelevante Vermeidung von ggf. stationären Untersuchungen in Narkose [1].

Das Otoskop ist ein Instrument, das von ca. 60 % der HNO-Ärzte nie eingesetzt wird. Die offensichtlichen Nachteile des Otoskops, also Monokularität, kurze Brennweite und die Blockierung einer Hand und die offensichtlich ubiquitäre Verfügbarkeit binokularer Mikroskope in Praxen und Kliniken könnte dies erklären. Häufiger nehmen klinisch als ambulant tätige HNO-Ärzte die Nachteile des Otoskops zumindest situativ in Kauf, möglicherweise zur Durchführung von konsiliarischen Untersuchungen bei immobilen Patienten. Wir finden darüber hinaus einen Unterschied zwischen jüngeren HNO-Ärzten, die das Otoskop häufiger einsetzen, im Vergleich zu älteren HNO-Ärzten [3]. Bei Pädiatern und Allgemeinärzten ist dagegen die Ohruntersuchung mit dem Otoskop Standard [3].

Der Umfang unserer Befragung mit 321 Teilnehmern war relativ gering verglichen mit der Gesamtzahl von etwa 5000 in Deutschland klinisch und ambulant tätigen HNO-Ärzten. Die für die eingeschränkte Weiterleitung der Fragebögen verantwortlichen Datenschutz-Probleme wurden dargelegt. Zumindest geografisch scheint eine Selektion unwahrscheinlich, da der Rücklauf aus den verschiedenen Bundesländern repräsentativ war und daher ein regionaler Vergleich möglich. Ob der hohe Anteil leitender Klinikärzte an der Studie die Repräsentativität der Angaben zu den angewandten Untersuchungstechniken erhöht, muss offen bleiben.

## Fazit für die Praxis

- Die klinische HNO-Untersuchung in Deutschland wird mit einer Stirnlampe in den neuen Bundesländern häufiger ohne Kabel durchgeführt.
- Der Stirnreflektor spielt mit weiter rückläufiger Tendenz kaum noch eine Rolle.
- Die Diagnostik der Ohren erfolgt fast immer mit einem Mikroskop, die übrigen Organe werden regelmäßig auch mit einem starren Endoskop untersucht.
- Auf deutlich niedrigerem Niveau sind flexible Endoskope und Otoskope bei jüngeren, klinisch tätigen Kollegen beliebter.
- Eine konsequente Nutzung des diagnostischen Potenzials der flexiblen Endoskopie des Larynx (und Epi‑/Hypopharynx) wird durch die ambulanten Vergütungsstrukturen in Deutschland möglicherweise kompromittiert.
